# Move & Improve: A Worksite Wellness Program in Maine

**Published:** 2006-06-15

**Authors:** Michele Polacsek, Liam M O’Brien, Wendie Lagasse, Nicole Hammar

**Affiliations:** Maine–Harvard Prevention Research Center, Maine Center for Public Health; Department of Mathematics, Colby College, Waterville, Me; Community Wellness Service, Eastern Maine Medical Center, Bangor, Me; Community Wellness Service of Eastern Maine Healthcare Systems, Brewer, Me

## Abstract

**Background:**

We describe the evaluation process and outcomes of Move & Improve, a worksite wellness program in Maine. The evaluation process was based on the Centers for Disease Control and Prevention's *Framework for Program Evaluation in Public Health* and community-based participatory research principles. Innovative approaches are required to address burgeoning chronic disease trends and risk factors. Worksites are an ideal setting in which to affect working adults and high-risk individuals. Using community-based participatory research methodology increases community capacity for evaluation, dissemination, and use of evaluation results.

**Context:**

Move & Improve is an ongoing program that was implemented in 1996. Although evaluation data have been collected since the program's inception, a more systematic evaluation based on community-based participatory research principles was undertaken in 2003 and 2004 with the technical assistance of the Maine–Harvard Prevention Research Center and Colby College.

**Methods:**

The Maine–Harvard Prevention Research Center facilitated the development of a program logic model, evaluation questions, data collection instruments, an analysis plan, presentations, and reports. We used a cross-sectional study design with nonparticipant comparison groups.

**Consequences:**

Data indicate possible program improvement strategies and substantial improvements in lifestyle factors among participants.

**Interpretation:**

Limitations of the evaluation include participant self-selection, cross-sectional study design, a lack of adequate resources for evaluation, and the challenges of using community-based participatory research methods. Despite these limitations, Move & Improve program staff consider the evaluation of the program a success and have learned ways to improve the program and future evaluation efforts. Overall satisfaction with the process has been nurtured through community-based participatory research methods. This approach also enabled us to meet key evaluation standards.

## Background

Health statistics for both Maine and the United States (1) underscore the need for reducing risk factors associated with cardiovascular disease, diabetes, heart disease, cancer, and obesity. Worksites offer ideal settings for reaching adults, including those at higher risk for chronic diseases. Worksite health promotion programs benefit employees and the organization (2-6). A recent review of worksite health promotion programs recommends that reporting on outcomes for these programs include more information about enrollment, implementation and maintenance, and negative outcomes (2).

Move & Improve, a worksite wellness program in Maine, was implemented in 1996 to encourage employees and community members to increase their physical activity. We describe the evaluation process implemented in 2003 and the program outcomes for 2003 and 2004. The Maine–Harvard Prevention Research Center (M-HPRC) assisted with the evaluation design and implementation. The program evaluation is based on the Centers for Disease Control and Prevention's (CDC's) *Framework for Program Evaluation in Public Health* (7) and community-based participatory research (CBPR) principles. The evaluation was designed and implemented with minimal resources.

M-HPRC's approach to community-based program evaluation is rooted in the literature on community organization and community building (8-10). This approach is consistent with models for data action research (11), research translation (12), and community engagement (13). CBPR is an approach to public health research that involves community members as equal partners. CBPR is 1) a participatory process in which power is shared and local expertise is recognized; 2) a cooperative process to which community members and researchers contribute equally; 3) a colearning process for researchers and community members; 4) a process that involves systems development and local community capacity building; 5) a process that empowers participants to increase control over their lives; and 6) a process that balances research and action (14).

## Context

Eastern Maine Medical Center established the Move & Improve program in 1996 to motivate individuals to increase their physical activity and to make healthier lifestyle choices. Move & Improve became a program of Eastern Maine Healthcare Systems in 2004. Move & Improve is a free 12-week program beginning in March each year that is designed to improve health by reducing participants' risk of chronic diseases and obesity. Individuals become involved with the program primarily, though not exclusively, through affiliations with worksites. Other Move & Improve sites include schools and community organizations. Yearly recruitment efforts include reaching out to past and new participants through the mail, statewide newsletters, and collaborating partners statewide who promote the program locally. Under the guidance of volunteer site coordinators who are identified internally by worksites, participants are asked to engage voluntarily in at least 30 minutes of physical activity for at least 4 days per week for a minimum of 8 weeks of the 12-week program. Participants are asked to track their physical activity on a log (either on paper or through an interactive online activity log) and receive encouragement and tips for continued participation and physical activity throughout the program. In addition, the program offers participants community-based stretch breaks at the local mall, statewide monthly walking clinics or clubs, various exercise programs, physical fitness assessments, educational sessions, and other events.

Various communication tools have been used over the past 8 years to convey helpful information to participants. These include a quarterly newspaper that features a tip of the week, good-for-you recipes, nutritional information, book reviews, and profiles of program participants and a weekly online newsletter. Move & Improve health promotion tips are shared communitywide and statewide through local newspapers (*Bangor Daily News*), the local CBS affiliate (WABI–TV 5), collaborating partners (i.e., Eastern Maine Medical Center, Sebasticook Valley Hospital, Inland Hospital, The Aroostook Medical Center, and the Healthy Hancock coalition), various program sponsors, Move & Improve coalition members, the Move & Improve Web site, and the e-mail systems of some participating worksites.

Move & Improve has collected data about the program since its inception. The number of participating individuals, participating sites, the number of individuals completing the program, and postprogram stage of change have been tracked since 1997. During the first 7 years, the program offered a paper-and-pencil evaluation to participants and site coordinators. In the eighth year, Move & Improve began offering all participants and coordinators an online evaluation.

Program participation and completion rates have continued to increase. In 1997, approximately 1000 participants registered for the program; in 2003, Move & Improve had more than 9000 participants, and in 2004 it had more than 11,000 participants. In 2003, almost half were first-time participants, and in 2004, approximately one third were first-time participants. In 2003, physical activity stage of change was measured using a five-stage algorithm (precontemplation, contemplation, preparation, action, and maintenance) based on exercising 4 days per week for 30 minutes per day, adapted from previous stage-of-change research (15). In 2004, however, Move & Improve staff became interested in describing participants' physical activity in greater detail and adopted a new measure of physical activity stage of change (using the same five-stage algorithm) which included an additional goal of exercising 5 days per week for 45 minutes per day. The program used current guidelines published by the American College of Sports Medicine to establish the goal levels (available from www.acsm.org). In both 2003 and 2004, a general forward movement through the stages of change was observed.

Since the program's inception, participants have reported anecdotally positive effects, such as weight loss, reduced stress, and reduced absenteeism. A technical report compiled by the University of Maine in 2001 (16) reported statistically significant differences in mean systolic blood pressure, total cholesterol, the ratio of total cholesterol to high-density lipoproteins, number of sit-ups in 1 minute, number of push-ups in 1 minute, and 3-minute recovery heart rate between pretest and posttest scores among a self-selected group of participants.

In early 2003, the Move & Improve program director approached the M-HPRC to help improve the evaluation design and process with the ultimate goal of contributing to general knowledge about worksite health promotion programs such as this one. The M-HPRC contributed some staff time and a small stipend to help with data analysis. A statistician from Colby College was engaged to help with data entry and analysis. M-HPRC and Colby College used principles of CBPR for carrying out this phase of the evaluation.

The objectives of this phase of the evaluation were to 1) characterize Move & Improve participants and participation in the program; 2) learn which worksite and coordinator policies seemed to make a difference to participants; 3) explore whether physical activity and stage of change were affected; 4) learn whether participants experienced other lifestyle outcomes such as weight change, better nutrition, stress reduction, or reduced absenteeism; and 5) explore whether multiyear participation was more likely to sustain greater levels of physical activity.

## Methods

M-HPRC staff led discussions outlining Move & Improve evaluation questions and methodology and were available for technical assistance throughout the process. M-HPRC staff also facilitated the development of a program logic model outlining the program's major activities and desired outcomes. M-HPRC staff facilitated the articulation of the program evaluation questions and evaluation design through negotiation with the Move & Improve staff who would implement it with minimal time and resources. M-HPRC staff drafted the posttest participant and coordinator surveys, which were then discussed and revised with Move & Improve staff input. Initial results were drafted by M-HPRC and Colby staff and presented to program staff for interpretation and revision. Because this evaluation process was community driven, Move & Improve staff made all final decisions about evaluation methodology based on discussions with M-HPRC and Colby staff and available resources.

The 2003 and 2004, evaluations used a cross-sectional study design. Both years' evaluation efforts included nonparticipant comparison groups. In 2003, the comparison site survey was conducted at a worksite in Maine among program participants and nonparticipants and at a nonparticipating corporate partner worksite in Vermont that had comparable demographics. Both comparison groups were suburban, and each had approximately 100 employees. However, the Move & Improve participant group was 63% female, whereas the combined nonparticipant comparison group was 86% female. The comparison groups were also slightly younger. The 2004 program identified a comparable comparison group (from a worksite employing more than 750 people) near the program office in Bangor, Me. Program staff decided to offer a pencil-and-paper format for the comparison group survey in 2004 rather than an online format, which the participants used.

A posttest survey was developed to assess participant demographics, level of physical activity, physical activity stage of change before and after participation, change in other lifestyle factors, absenteeism, and years of participation. One thousand randomly selected participants were mailed surveys in 2003 within 1 week of program completion. In 2004, all participants were provided with an opportunity to fill out an online evaluation that followed participation in the program.

In 2003, coordinator surveys were developed and mailed to all program coordinators. In 2004, coordinators were offered a survey online. The surveys assessed coordinator demographics, level of physical activity, and strategies coordinators used to motivate participants.

Because the information gathered from program participants was generally categorical, hypothesis testing to find associations between them was done using contingency table analyses. Healthy living indicators (e.g., fat intake, soft drink consumption, fruit and vegetable consumption) were recorded as having increased, decreased, or not having changed — yielding three nominal categories for each indicator. Change in stage of change was calculated as being the final stage of physical activity (postprogram) minus the initial stage of physical activity (preprogram). Thus, a negative stage of change indicates a decrease in physical activity, and a positive stage of change indicates an increase in physical activity. A three-level categorical variable was used to indicate whether each participant had a *decrease* in physical activity, *no change* in physical activity, or an *increase* in physical activity. This three-level outcome was used in place of the stage of change because of the large number of sparse cell sizes resulting from small numbers reporting very large changes. As expected, few participants selected maintenance (which requires 6 months or more of consistent behavior) or precontemplation (which suggests not yet intending to take action). Contingency table analyses using the Fisher exact test were used to assess the strength of the association between this three-level measure of change in physical activity and the lifestyle factors about which information was obtained. We also explored where movement in stage of change tended to take place between preprogram and postprogram, stratifying by stage. Preprogram stage was determined by participants' recall at the end of the program.

## Consequences

Approximately 43% of participants in 2003 completed the program, and approximately 46% completed the program in 2004, the highest percentage thus far. Of the participants who completed the program in 2003, 317 (31%) responded to the evaluation; in addition, 177 (53%) of the site coordinators, 33 (83%) of the individuals from the in-state comparison site, and 40 (80%) from the out-of-state comparison site responded to the evaluation. In 2004, 902 (14%) of the 6291 participants who completed the program responded to the online evaluation; in addition, 139 (39%) of 355 site coordinators and 252 (34%) of the 750 eligible employee nonparticipants completed surveys in 2004. Participants from both years were predominantly female (87% in 2003 and 75% in 2004). The substantial decrease in the proportion of female participants in 2004 may be because of a significant program effort in that year to recruit more male participants. The most frequent participant age categories were 45 to 49 years and 50 to 54 years for both years, with approximately 20% of participants in those categories (Table). Age and sex of participants and nonparticipant comparisons were similar (data not shown). Participation in worksites varied greatly and ranged from as little as 15% to more than 50% in some worksites. Two hundred and seventy worksites participated in 2003, and 294 participated in 2004.

**Figure 1:**
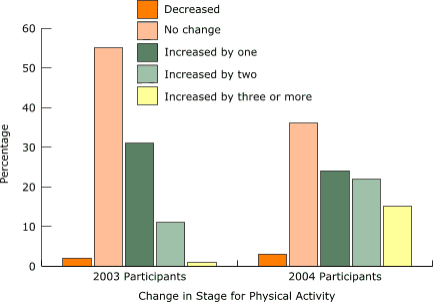
Percentage of Move & Improve participants in each category of change for physical activity at posttest, 2003 and 2004.

Both years of data show that in the 3 months before participating in Move & Improve, more than half of all participants reported no regular exercise or only minimal exercise. As expected, the posttest data from both years show substantial increases in physical activity, with only about 5% of participants remaining inactive or minimally active both years, compared with 27% of comparison group nonparticipants in 2004. In 2004, 61% of all participants increased their physical activity stage of change by one stage or more, and 37% had an increase of two stages or more (Figure 1). In contrast, the majority of comparison group nonparticipants did not increase their stage of physical activity during the same period. Participants beginning in the contemplation stage were more likely to move two stages or more than those beginning in later stages of physical activity (84% in 2004). In 2004, participants beginning in stages 4 and 5 were most likely to report no change (69% of participants in stage 4 and 63% in stage 5). Results were similar for 2003. The lack of movement in later stages is evidence of a ceiling effect: participants who were already more physically active at the beginning of the program were more likely to maintain the same physical activity levels.

**Table:** 

**Figure 2:**
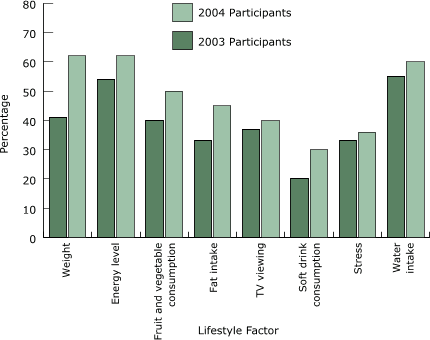
Percentage of Move & Improve participants with positive change in lifestyle factors at postprogram, 2003 and 2004. All factors were significantly associated with forward movement in physical activity stage of change at *P* < .001 in both years, except for sugar-sweetened soft drinks (*P* = .08).

In both 2003 and 2004, a substantial proportion of program participants reported weight loss (41% in 2003, 62% in 2004); increased energy (54% in 2003, 62% in 2004); increased fruit and vegetable consumption (40% in 2003, 50% in 2004); decreased fat intake (33% in 2003, 45% in 2004); decreased television viewing (37% in 2003, 40% in 2004); decreased sugar-sweetened soft drink consumption (20% in 2003, 30% in 2004); decreased stress (33% in 2003, 36% in 2004); and increased water intake (55% in 2003, 60% in 2004) (Figure 2). Each of these improved lifestyle factors was significantly associated with participants' forward movement in physical activity stage of change (*P* < .001) except for soft drink consumption (*P* = .08). These results are particularly impressive given that the ceiling effect would likely bias the results toward the null of no association.

**Table:** 

Employee absenteeism seemed to be associated with improvement in stage of change. Employees with a forward movement of two or three stages averaged 1 sick day during the 3 months of the program, whereas employees who stayed the same or regressed in their stage of change averaged 1.5 sick days during the same 3 months in 2003. However, number of sick days reported was small, so we were not able to assess significance. Involvement with Move & Improve for 2 or more years was significantly associated with improvement in stage of change (*P* = .02).

Flextime, group activities, and incentives were the most common practices perceived by participants to make a difference. Coordinators cited group activities and incentives as worksite policies that came about most often as a result of the program. Yet, in contrast to what participants noted would be most helpful, coordinators tried to motivate participants most often using posters, office memos, wellness committees, bulletin boards, and e-mail tips and by registering employees for them.

## Interpretation

We evaluated Move & Improve's process and outcomes using CBPR methods. Limitations in our ability to draw conclusions about the program's success include limitations inherent in cross-sectional study design; the small size and location of the comparison groups; the lack of program resources, including staff and funds for long-term follow-up with participants; and challenges that arise from using CBPR methods and local decision making. Move & Improve participants are self-selected and therefore do not represent the general worksite population or any particular high-risk group. Because participants' stage at the start of the program was determined by recall at the end of the program, response bias may have slightly inflated the effects of the program as reported in 2003 and 2004.

Move & Improve's challenge now is to increase participation and completion rates and to recruit worksites with higher-risk adult populations. The 2004 evaluation revealed that participants felt that incentives, group activities, and flextime at work made a difference in their participation. Coordinators, however, may not have been able to influence worksite policies to include these factors. Because evaluation data revealed that younger individuals and men tended not to participate at as great a rate as relatively older individuals and women, greater efforts should be made by the program to help coordinators recruit and retain these individuals and to understand barriers to their participation. Perhaps more coordinators should fit this profile to motivate individuals from these groups to participate. Our analyses also revealed that many participants who were already physically active did not change their stage of physical activity over the course of the program. Perhaps some formative data could help elucidate the types of program components that may motivate these individuals to stay active over time. To motivate and recruit individual worksites to participate, program staff could emphasize the data that indicate less absenteeism for participants. Objective record keeping of numbers of sick days taken by participants should be encouraged.

Many final modifications to the evaluation design and instruments were made because of resource constraints by program staff after consultation with M-HPRC and Colby College. These circumstances may have compromised the scientific rigor of the study and our ability to draw objective conclusions from the data. One example is the decision to change the way physical activity stage of change, a key outcome, was measured in 2004. This decision was made by Move & Improve staff to align the outcome measure with the additional program goal of 5 days of physical activity per week for at least 45 minutes per day. Another example is the program's decision to offer paper-and-pencil surveys for the 2004 comparison site when all of the program participants had completed the same survey online. In this case, the employer preferred the pencil-and-paper format. Another modification involved the decision in 2003 to survey in-state nonparticipants for the comparison group from the same worksite as participants. This approach required fewer resources than going to another worksite. With a deeper understanding of the importance of consistency of measurement, Move & Improve program staff plan to use the original physical activity stage of change measure in the future and, because of staffing and funding constraints, discontinue the use of a comparison survey.

An advantage of using CBPR was that it increased our adherence to several core evaluation standards (7). The standard of *utility* was maximized by involving stakeholders so that identification of their needs and intent were not only addressed but were central to the process. Evaluator credibility was enhanced through the relationships that were nurtured. Any findings were first disseminated to key program stakeholders for their review and interpretation. The standard of *feasibility* was also positively affected through CBPR. Evaluation procedures had to be practical given the resources. Move & Improve staff made all final decisions on how to carry out the evaluation based on their assessment of whether they could get it done in a timely manner. Maximizing feasibility, however, may have also compromised scientific rigor.

Move & Improve program evaluation indicates that the program has been a success on many levels. Evaluation data indicate that Move & Improve has a significant impact on participants' lifestyle and risk behaviors and that longer participation in the program may also be associated with greater chronic disease risk reduction. Participants significantly increased their physical activity stage of change compared with nonparticipants during the same time period. Improved lifestyle factors were also significantly associated with forward movement in stage of change. Future evaluation efforts can minimize limitations by adding pretest data collection and keeping measures consistent over time. Longer-term follow-up of participants should also be attempted.

The benefits of using CBPR methodology far outweighed limitations in scientific rigor. Move & Improve staff enthusiasm, at least in part because of its integral involvement with every aspect of the evaluation design, helped Move & Improve and M-HPRC staff overcome key barriers. Program staff gained appreciation for how to carry out successful program evaluation with minimal resources and how to improve their program. Several core standards of successful evaluation practice were also maximized. Relationships formed through the process of conducting CBPR-framed evaluation will help to sustain and improve future Move & Improve program evaluation efforts.

## Figures and Tables

**Table.** Age and Sex of Participants in Move & Improve, Maine, 2003 and 2004

**Characteristic**	**2003, % (n = 317 )**	**2004, % (n = 902)**
**Age, y**		
18-20	0	<1
21-24	2	3
25-29	5	5
30-34	8	10
35-39	7	11
40-44	15	17
45-49	20	18
50-54	18	18
55-59	11	11
60-64	6	4
65-69	5	1
≥70	3	<1
No response	0	1
**Sex**		
Female	87	75
Male	13	23
No response	0	2

## References

[1] (2002). Centers for Disease Control and Prevention (CDC). Behavioral Risk Factor Surveillance System survey data.

[2] Bull SS, Gillette C, Glasgow RE, Estabrooks P (2003). Work site health promotion research: to what extent can we generalize the results and what is needed to translate research to practice?. Health Educ Behav.

[3] Ostwald SK (1989). Changing employees' dietary and exercise practices: an experimental study in a small company. J Occup Med.

[4] Heirich MA, Foote A, Erfurt JC, Konopka B (1993). Work-site physical fitness programs. Comparing the impact of different program designs on cardiovascular risks. J Occup Med.

[5] Lechner L, de Vries H, Adriaansen S, Drabbels L (1997). Effects of an employee fitness program on reduced absenteeism. J Occup Environ Med.

[6] Poole K, Kumpfer K, Pett M (2001). The impact of an incentive-based worksite health promotion program on modifiable health risk factors. Am J Health Promot.

[7] (1999). Framework for program evaluation in public health. MMWR Recomm Rep.

[8] Minkler M, Wallerstein N, Glanz K, Lewis FM, Rimer BK (1997). Improving health through community organization and community building. Health behavior and health education: theory, research, and practice.

[9] Bracht NF (1998). Use of community analysis methods in community-wide intervention programs. Scand J Prim Health Care Supp.

[10] Green L, Daniel M, Novick L (2001). Partnerships and coalitions for community-based research. Public Health Rep.

[11] Israel BA, Schulz AJ, Parker EA, Becker AB (1998). Review of community-based research: assessing partnership approaches to improve public health. Annu Rev Public Health.

[12] Green LW, Johnson JL (1996). Dissemination and utilization of health promotion and disease prevention knowledge: theory, research and experience. Can J Public Health.

[13] (1997). CDC/ATSDR Committee on Community Engagement. Principles of community engagement.

[14] Minkler M, Wallerstein N, Minkler M, Wallerstein N (2003). Introduction to community based participatory research. Community-based participatory research for health.

[15] Polacsek M, Rogers EM, Woodall WG, Delaney H, Wheeler D, Rao N (2001). MADD victim impact panels and stages-of-change in drunk-driving prevention. J Stud Alcohol.

[16] Cyr N (2001). Eastern Maine Medical Center's Move & Improve Program: community wellness program research data analysis technical report.

